# Literature Review on Artificial Intelligence Methods for Glaucoma Screening, Segmentation, and Classification

**DOI:** 10.3390/jimaging8020019

**Published:** 2022-01-20

**Authors:** José Camara, Alexandre Neto, Ivan Miguel Pires, María Vanessa Villasana, Eftim Zdravevski, António Cunha

**Affiliations:** 1 R. Escola Politécnica, Universidade Aberta, 1250-100 Lisboa, Portugal; 1701367@estudante.uab.pt; 2 Instituto de Engenharia de Sistemas e Computadores, Tecnologia e Ciência, 3200-465 Porto, Portugal; alexandre.h.neto@inesctec.pt; 3 Escola de Ciências e Tecnologia, University of Trás-os-Montes e Alto Douro, Quinta de Prados, 5001-801 Vila Real, Portugal; impires@it.ubi.pt; 4 Instituto de Telecomunicações, Universidade da Beira Interior, 6200-001 Covilhã, Portugal; 5 Centro Hospitalar Universitário Cova da Beira, 6200-251 Covilhã, Portugal; 72152@chbv.min-saude.pt; 6 UICISA:E Research Centre, School of Health, Polytechnic Institute of Viseu, 3504-510 Viseu, Portugal; 7 Faculty of Computer Science and Engineering, University Ss Cyril and Methodius, 1000 Skopje, North Macedonia; eftim.zdravevski@finki.ukim.mk

**Keywords:** eye diseases, glaucoma screening, artificial intelligence, deep learning, image processing, glaucoma classification, mobile devices, digital camera

## Abstract

Artificial intelligence techniques are now being applied in different medical solutions ranging from disease screening to activity recognition and computer-aided diagnosis. The combination of computer science methods and medical knowledge facilitates and improves the accuracy of the different processes and tools. Inspired by these advances, this paper performs a literature review focused on state-of-the-art glaucoma screening, segmentation, and classification based on images of the papilla and excavation using deep learning techniques. These techniques have been shown to have high sensitivity and specificity in glaucoma screening based on papilla and excavation images. The automatic segmentation of the contours of the optic disc and the excavation then allows the identification and assessment of the glaucomatous disease’s progression. As a result, we verified whether deep learning techniques may be helpful in performing accurate and low-cost measurements related to glaucoma, which may promote patient empowerment and help medical doctors better monitor patients.

## 1. Introduction

In addition to today’s discussions related to problems in the doctor–patient relationship and the deficiency of clinical examination that makes the diagnosis more dependent on complementary tests in the context of public health [1], new problems are emerging related to the use of new technologies to support medical diagnoses [2,3,4,5]. These issues can be associated with the security of electronic medical records, the exponential increase in the production of new data arising from these new technologies, and how these data will be processed [6,7,8,9].

In one’s family history, glaucoma in first-degree relatives indicates an increased possibility of developing the disease compared to an adverse family history of illness [10,11]. One of the risk factors is systemic diseases, such as high blood pressure and diabetes, rheumatic diseases, autoimmune diseases, and the use of steroids which can be predisposing factors for the development of the disease [12]. In addition, eye diseases, such as cataracts, tumors, inflammatory processes, trauma, and ocular hypertension can also be risk factors for glaucoma [12].

Considering the importance of vision in modern society, the disease at an advanced stage can potentially reduce one’s quality of life to different degrees, causing emotional and workforce damage and certainly a greater use of health resources [13]. The risk of blindness depends on susceptibility factors, such as family history, the way the disease progresses, the level of eye pressure and age at disease onset, previous eye diseases and injuries, topical and systemic use of corticosteroids, consumption of tobacco, alcohol, and drugs, diabetes, lung disease, heart disease, cerebrovascular disease, and high blood pressure [14,15,16]. Treatment is based on clinical and surgical strategies to reduce intraocular pressure, the only factor susceptible to change [17,18]. In addition, it aims to reduce the progression of optic nerve damage and maintain vision for a more extended period of time [19,20].

However, the patient’s record and the storage and sharing of medical data and images must be well protected [9]. Therefore, electronic health records require precautions that include controlling access to data, the insertion and deletion of information, storage, and the transmission of data and images [21,22,23].

Artificial intelligence (AI) algorithms tend to improve the processing of this large amount of data and propose increasingly accurate diagnostic hypotheses [4,24,25]. AI is sometimes heralded as the new industrial revolution [8,24]. Deep learning (DL) is the steam engine of this revolution which can process a large amount of data that operates data analysis by representing successive layers inspired by the human brain [25].

DL started in 1943 and emerged with the evolution of neural networks which became deeper with new layers in a subset of AI [26]. In 2012, it became more popular with the ImageNet Large-Scale Visual Recognition Challenge (ILSVRC), and the scientific community adopted it. It yields results which are close to state of the art or better in many areas [27]. These technologies are used in image recognition, real-time translations, voice recognition services such as Siri from Apple, Alexa from Amazon, and Cortana from Microsoft [28,29]. In addition, multiple studies have shown that DL algorithms are state of the art in breast histopathology analysis, skin cancer classification, risk of cardiovascular disease prediction, the detection of lung cancer, and several applications in the ophthalmic area with the potential to revolutionize the diagnosis of eye diseases [30,31,32].

DL has become an essential tool for interpreting data derived from digital photos, optical coherence tomography, and visual field, contributing to tracking diseases such as senile macular degeneration, diabetic retinopathy, and glaucoma [33,34,35]. In Canada, it is used for teleophthalmology to screen populations that live in more distant regions, improve access to under-served communities, and alleviate the shortage of doctors [36,37,38].

In ophthalmology, DL algorithms have shown their enormous potential for screening and diagnosing pathologies such as diabetic retinopathy and glaucoma through the analysis and processing of retinal images and problems related to the prediction of daily pressure changes in the eye [39,40,41,42,43].

This study aimed to perform a literature review of artificial intelligence and DL methods for glaucoma screening, segmentation, and classification. This was included in a project for glaucoma screening and classification to be implemented in a system that will be easy to use for the patients, promoting patient empowerment with these tools [44], enabling the healthcare professional to monitor the evolution of glaucoma disease via remote consultation. Medicine is a vast science, and the team has different projects related to various fields [45,46,47,48,49], including ophthalmology.

This study shows that different databases containing retina images are available that allow the research and creation of the automatic method. However, it was only initially used because medical people are involved in this project to provide accurate data for the study of glaucoma disease. Furthermore, this study also reviews the methods for the segmentation and classification of glaucoma disease by images.

This paragraph ends the introductory section. Section 2 will then present some public databases that can be used for glaucoma screening and classification. Furthermore, some studies related to DL to analyze glaucoma are presented in Section 3. Sequentially, Section 4 outlines the segmentation methods for this study. In addition, Section 5 presents the use of classification methods, followed by the discussion of the combination of the different methods in Section 6. Finally, Section 7 concludes this review.

## 2. Public Databases

Public databases contain eye images for study, research, and methodological standardization [50]. Databases of public photos can be obtained for different nationalities, from multiple patients, in different locations, by different cameras, which can vary in size, in the centralization of the focus of interest, and the delimitation of the area of interest [51]. The multiplicity of conditions under which photos are taken in different places using cameras with different settings, taken from various groups of patients, with objectives defined by multiple specialists, can interfere with the results [52]. The databases for the study of pathologies of the posterior pole of the eye show the retina, vessels, macula, and optic disc [53]. Other image databases for the study of glaucoma centered on the optic papilla with demarcations of the papilla and excavation boundaries made by specialists represent the gold standard, serve as reference standards for segmentation measurements, and can exploit the implementation of DL networks [54].

The authors of [55] identified 94 open databases containing 507,724 ophthalmic images and 125 videos from 122,364 patients. These databases are often used in various eye research studies using machine learning. However, few public fundus image sets for evaluating glaucoma contain classification into groups of normal papillae and disease evolutionary stages, the marking of optic disc segmentation, and excavation in both eyes.

Several databases are frequently used to classify and segment the optical disc and for excavation in automated systems for detecting optical papillary features. One of them is RIM-One, an open retinal image database for optic nerve evaluation [56]. The manual segmentation of each image is used to form the gold standard obtained for each image by centralizing all contours and reference points, eight axis directions from one reference point, and the insertion axes of the shapes are delineated in each order of each of the five manual contours. Intersections and reference points are computed at five distances in each direction and form a radial average. The RIM-One database photographs are accessible at [57] and consist of three sets of pictures, such as RIM-One-r1, RIM-One-r2, and RIM-One-r3, published in 2011, 2014, and 2015, respectively.

RIM-One-r1, published in 2011, is a database for evaluating, locating, and detecting the optic disc formed by retinal images obtained from 118 healthy eyes and 51 patients with various stages of glaucoma through a Nidek AFC-210 non-mydriatic camera with Canon EOS 5D Mark II 21.1 megapixels with 45 degrees horizontal and vertical field [56]. In addition to diagnosis, five specialists also include the manual segmentation of the optic disc. The patients were from three Spanish hospitals: Hospital Universitario de Canarias (HUC) in Tenerife; Hospital Universitario Miguel Servet (HUMS) in Zaragoza; and Hospital Clínico Universitario San Carlos (HCSC) in Madrid.

RIM-One-r2, published in 2014, is an extension of the first version having some duplicated images, containing 255 photographs of healthy eyes and 200 photos of patients with glaucoma at HUC and Hospital Universitário Miguel Servet using the same cameras as the previous version. Furthermore, medical experts manually segmented images [56,58].

RIM-One-r3, published in 2015, contains 85 images of healthy eyes (without glaucoma) and 74 images of patients with glaucoma [34]. The main difference between this and previous versions is that the images were exclusively obtained at the HUC with a Kowa WX 3D non-mydriatic camera [56]. The images were centered on the optic nerve head using a 34-degree angle, obtaining horizontal stereoscopic images of 20 degrees of vision and a vertical sun of 27 degrees with a total resolution of 2144 × 1424 pixels per image. Stereoscopic photographs were submitted to manual segmentation of the optic disc E of the excavation by two specialists, and some photos are contained in RIM-One-r2.

RIM-ONE DL was created in 2020 to optimize the previous ones for use in DL. It consists of 313 normal and 172 glaucoma fundus from patients [59]. Two experts manually segmented all images (disc and excavation) again, and in cases of doubt, another specialist with 20 years of experience was consulted, who made the final decision. One image of each eye per patient was kept. All images were cropped around the optic nerve head using the same proportionality and stored in .png and the file names fixed in r1, r2, and r3 according to the RIM-version 1 that was extracted which made a clearer division between the training group (samples from the HUC) and test group (samples from the Hospitals of Madrid and Zaragoza). In the two groups, the manual segmentation of the disc and excavation were included and divided into two sets. The first set was divided into training and testing in a ratio of 70:30. Additionally, the second set from the HUC was used for training (195 normal and 116 with glaucoma). Finally, the images used from two other hospitals were divided (118 normal and 56 with glaucoma). Images were rescaled in intensity between 0 and 1 and submitted to several models with DL architecture: Xception, VGG16, VGG19, ResNet50, Inception-V3, InceptionResNetV2, MobileNet, DenseNet121, NASNetMobile, and MobileNetV2 resized to an 224 × 224 × 3 input layer. A 2D global average pooling layer was connected to two output layers using SoftMax to distinguish between normal and glaucoma classes with satisfactory results in and with maximum values obtained with the VGG19 network, revealing an AUC of 98.67% and an accuracy of 93.15% for the set of random tests, versus an AUC of 92.72% and an accuracy of 85.63% for the Madrid and Zaragoza test set.

DRISHTI-GS is a retinal image dataset for optic nerve head segmentation [60] developed and distributed to provide means for the evaluation of CAD systems in the detection of glaucoma, evaluated by four experts. It presents a set of retinal images for papilla evolution in normal and glaucomatous eyes with manual segmentation performed by specialists, which allows performing measurements of the diameter ratio and cupping and optic disc area, to establish the presence or absence of presence or absence or glaucoma, called DRISHTI-GS1 [61]. It consists of 50 training and 51 test images, in which the papilla was manually segmented by different experts. It is an extension of DRISHTI-GS. Regarding the lack of more comprehensive data, the author warns about the difficulty of comparing the performance of individual methods.

DRIONS-DB [62] is a public database for the comparative evaluation of optic nerve head segmentation from digital retinal images. The database is open to test algorithms, as well as compare and share results with other researchers. It contains 110 retinal images with 600 × 400 pixel resolution, with outer optical disk boundaries delimited by two medical experts using image annotation software [63]. Additionally, it includes images in digital format and scanned using HP-PhotoSmart-S20 high-resolution scanner, RGB format, 600 × 400 pixel resolution, and an 8 bits/pixel, centered on the optical disk [63].

Messidor database [64] contains hundreds of fundus images and has been publicly distributed since 2008. It was created by the Messidor project to assess the automatic targeting of retinal lesions and diabetic retinopathy [65]. It is an implementation program for testing segmentation algorithms. It contains 1200 color fundus images, 800 with pupil dilation and 400 without dilation. It is mainly used for the classification of diabetic retinopathy.

High-Resolution Fundus (HRF) contains 15 images from healthy patients, 15 images from glaucomatous patients, and 15 images from patients with diabetic retinopathy [66]. The photos have 3504 × 2336 pixel resolution with binary images of segmentation of vessels.

The Optic Nerve Head Segmentation Dataset (ONHSD database) comprises 99 fundus images from 50 patients of a variety of ethnic backgrounds (Asian, Afro-Caribbean, Caucasian) with the respective annotations of the optic disc made by clinical experts. The images were acquired with a field angle lens of 45 degrees and a resolution of 640 × 480 pixels [67].

ACRIMA database [68] contains 705 images, including 396 images of patients with glaucoma and 309 of patients without glaucoma, and does not include disc segmentation and excavation.

REFUGE database [69] contains 120 images of glaucoma patients and 1080 images of healthy patients with a segmented disc and cup, which were captured from different cameras and show a clear division between training and test data. There is a need to expand the number of public image data available that meet these requirements.

ORIGA database [70] comprises 168 images of patients with glaucoma and 482 photos of healthy patients. Its data include disc and cup segments. The problem with this database is that although it appears to have been public at one point, to the best of our knowledge, it stopped being public some time ago.

Sjchoi86-HRF database [71] has 601 fundus images in 4 subsets: normal (300 images); glaucoma (101 images); cataract (100 images); and retinal disease (100 images). The images were captured using 8 bits per color plane at 2592 × 1728, 2464 × 1632, or 1848 × 1224 pixels.

Other databases used in the literature include the Singapore Chinese Eye Study (SCES) database [72], composed of 1676 images with 46 glaucoma cases, and the Singapore Indian Eye Study (SINDI) [72], consisting of 5783 images of 113 eyes with glaucoma, which were used with the test.

Table 1 summarizes the characteristics of the presented public databases, where it is possible to verify that 50% of the analyzed databases contain images related to optic disc and cup, 40% of them did not include any of them, and 10% (one database) include only images related to the optic disc.

The public databases of optic papillary photographs constitute a reference standard that provides elements of analysis and differentiation from the normal optic papilla of glaucomatous eyes. However, DL methods have been highly representative of automated diagnoses. Nonetheless, they depend on a sufficient amount and variety of data to train and test the system as reference standards for comparison. Furthermore, it is observed that the standards offer images in different resolutions and formats and present a limited amount of data, increasing the difficulty of comparing them.

In [59], it is recommended that the public databases of retinal images meet the following requirements:

1. Availability of publicly accessible image sets, labeled by various experts, sufficient for use in DL methods;
2. Clear separation between training and testing sets to increase reliability between training and testing data;
3. Presence of diversity in the set of images, with variety meaning images captured by various devices involving patients of different ethnicities, and images captured in different conditions of lighting, contrast, noise, etc., in addition to having a preliminary diagnosis and including the segmentation of disc and cup manual reference.

The authors of [59] also made some criticisms of the other versions of RIM-ONE, including:

1. The combination of the other arrangements for DL problem solving and the indiscriminate variety of images from the three versions can lead to inconsistent results since different experts performed the segmentation;
2. Since RIM-ONE was not initially designed for DL, a clear division of training and testing images was never established;
3. Images were taken in different hospitals with different cameras, but only one camera was used in each version;
4. Only the r3 version had the cup segmented manually, and the previous versions only had the optic disc segmented. The experts involved in manual segmentation were not the same in all cases.

The RIM-One-r3 and DRISHTI-GS databases have an optical papilla centered and a periphery of approximately 30 degrees around the papilla that allow for the visualization of the excavation characteristics and sectorial defects in the fiber layers, an element to be considered in the classification of glaucoma initials.

Of all the databases mentioned, only the REFUGE and RIM-One DL databases meet the additional requirements of offering images from different cameras and a clear division of training and testing data. However, given the importance of the quantity and the representativeness of data required in automated systems, we cannot abandon the various optical papilla images contained in other databases, even if they do not meet all the requirements to be used in DL methods.

There seems to be a clear need to standardize and expand the number of public image databases that meet all these requirements and add the most significant data containing the greatest number of optic papillary region changes.

## 3. Deep Learning Methods

The evolution of glaucomatous neuropathy leads to an enlargement of the cup (inner part) compared to growth of the optic disc (outer part). The region between the cup and the outer limit of the papilla is called the neural rhyme. It comprises approximately 1,200,000 ganglion cell fibers from the retina and travels along the optic nerve carrying visual information to the brain’s occipital lobe, where it will be interpreted.

The following are measures for evaluating glaucomatous papillae:

1. The ratio between the cupping and the total vertical or horizontal diameter of the papilla or cup-to-disc ratio (CDR) indicates the presence of glaucoma if it has high values;
2. The ratio of the cup area to the papilla area, also called the cup-to-disc area ratio (CDAR);
3. Inferior, superior, nasal, and temporal (ISNT) rule describes a feature of the healthy optic disc thicker in the inferior pole, followed by superior, nasal, and temporal ones. When this sequence is altered (in this order), either by a change in diameter or area, it can be an early sign of injury. Optical Disc Damage Probability Scale or Disc Damage Likelihood Scale (DDLS) is based on the probability of optic disc damage by comparing the neural rim diameter with the optic disc diameter and the shortest distance between the optical disc contour and excavation.

The automated methods using DL techniques were grouped into methods that were based on:

1. Appearance or glaucoma screening (Section 3.1);
2. The segmentation of the outer limits and measurement calculations of optic disc structures (Section 3.2);
3. The segmentation of the outer limits of the optic disc and the excavation by detecting glaucomatous features of the papilla (Section 3.3);
4. Identification of early forms of glaucoma by CNN through fiber layer defects (Section 5).

### 3.1. Glaucoma Screening

Glaucoma screening is very ponderous since it only has symptoms when the disease is quite advanced. Therefore, the precocious diagnosis of this disease is essential. The glaucoma screening with digital fundus photograph (DFP) is a non-invasive method which suitable for large-scale screening. An automated system can decide whether there are any signs of glaucoma. For glaucoma screening based on DFP with disc parametrization, the optic disk and cup regions are segmented for the future evaluation of disc parameters. The optic disc appears as a bright circular or elliptical area, partially occluded by blood vessels, and the retinal nerve fibers converge to the optic disc and form the region called the cup. After the optic disc and cup segmentation, the cup-to-disc ratio can be calculated and used to estimate glaucoma. Glaucoma can be detected through automatic classification algorithms that learn features from DFP-labeled images [73].

Automated classification methods allow the glaucomatous papilla to be detected by its appearance [74], improving results and enabling mass tracking. However, the authors of [31] warn that non-segmented methods based on image characteristics need a large dataset for learning DL networks.

Regarding glaucoma screening, several studies have been performed to avoid blindness. The authors of [74] developed a diagnostic method for glaucoma using an image automation procedure obtained by relatively inexpensive cameras and automatic feature extraction by retinal image segmentation to analyze its geometric features. The study differs from other state-of-the-art methods by analyzing the glaucomatous characteristics of the papilla used to train the net—namely the diameter and area of the disc and cup—obtaining high accuracy in training under real conditions. It concludes that even in the images obtained at high resolution, there was no significant increase in accuracy experiencing neural rhyme variations as a database. However, the performance is lower in the photos with lower resolution, and the results seem to improve by adding features’ appearance geometrics.

The authors of [75] used several retinal fundus images available in ORIGA, RIM-One-r3, and DRISHTI-GS databases to fuse the results of different deep learning networks to classify glaucoma evolution. It shows that the results are promising, reporting an AUC of 94%.

In [76], the authors presented the DeepLabv3+ and MobileNet methods for the optic disc segmentation, and it was tested with RIM-ONE, ORIGA, DRISHTI-GS, and ACRIMA databases. The obtained results are reliable, presenting an accuracy of 97.37% (RIM-ONE), 90.00% (ORIGA), 86.84% (DRISHTI-GS), and 99.53% (ACRIMA). Additionally, the method presents an AUC of 100% (RIM-ONE), 92.06% (ORIGA), 91.67% (DRISHTI-GS), and 99.98% (ACRIMA).

The authors of [77] used a modified U-Net architecture with SE-ResNet50 for the segmentation of optic disc and optic cup for glaucoma diagnosis. They used DRISHTI-GS, REFUGE, and RIM-One-r3 databases to test the methods. They used DRISHTI-GS, REFUGE, and RIM-One-r3 databases to test the methods. The average results reported by the implemented method consisted of an AUC of 94%, showing the method’s robustness.

In [78], the authors implemented a direct cup-to-disc ratio (CDR) estimation method composed of an unsupervised feature representation of a fundus image with a convolutional neural network (MFPPNet) and a CDR value regression by random forest regressor. They used two datasets, namely Direct-CSU and ORIGA, for the testing and validation, reporting an AUC of 0.905.

The authors of [79] presented a fuzzy broad learning system-based technique for optic disc and optic cup segmentation with glaucoma screening, implementing it with the RIM-One-r3 dataset and SCRID dataset from the Shanghai Sixth People’s Hospital. It reports an AUC of 90.6% (RIM-One-r3) and 92.3% (SCRID).

In [80], a deep learning method was implemented for glaucoma screening featuring CDR and optic disc regions. They used different types of networks, including ResNet and segmentation network (SegNet), to calculate the vertical CDR. Additionally, as the leading architecture, it implements the U-Net, reporting a minimum DICE value of 89.6% and a precision of 95.12%.

The authors of [81] implemented a deep learning method with a binary classifier for glaucoma screening with a dataset of 5716 images from Asian and Caucasian populations. The results reported an AUC of 94% in images with detectable glaucoma.

In [82], a platform was designed for glaucoma screening that implements deep learning techniques for glaucoma diagnosis. The authors implemented mathematical models and a threshold classifier with 933 healthy and 754 glaucoma images, reporting a sensitivity of 73% and a specificity of 83%.

The authors of [83] used the M-Net method to segment the optic disc and optic cup tested with the REFUGE dataset for glaucoma screening. The results reported a DICE coefficient of 94.26% for the optic disc and 85.65% for the optic cup. Additionally, it reports an AUC of 96.37% and a sensitivity of 90%.

In [84], the HRF database was used to test the DL-ML hybrid model for glaucoma screening in 30 images. The results reported an accuracy of 100% and a sensitivity of 1.0. The system is reliable to help medical doctors in the diagnosis of glaucoma.

The authors of [85] implemented the patch-based deep network (GlaucoNet) framework to perform glaucoma screening with the DRISHTI-GS, RIM-ONE, and ORIGA datasets. The results reported an overlapping score of 91.06% (DRISHTI-GS), 89.72% (RIM-ONE), and 88.35% (ORIGA) for optic disc segmentation, and 82.29% (DRISHTI-GS), 74.01% (RIM-ONE), and 81.06% (ORIGA) for optic cup segmentation.

In [86], a deep ensemble network with an attention mechanism for the detection of glaucoma with optic nerve head stereo images was implemented. It consists of a convolutional neural network and an attention-guided network. The authors used a stereo glaucoma image dataset from Tan Tock Seng Hospital, Singapore. It comprised 282 images with 70 glaucoma cases and 212 normal cases. The results reported a sensitivity of 95.48%.

The summarization of the automated DL methods that help perform glaucoma screening is presented in Table 2.

### 3.2. Segmentation of the Outer Limits and Measurement Calculations of Optic Disc Structures

The segmentation of the outer limits is one of the techniques for detecting and analyzing glaucoma in images. It allows the measurement calculations of the optic disc structures.

In [87], the authors found that the detection methods of optic disc abnormalities result in the complex analysis of algorithms, operating costs, and dependence on vessel segmentation. In addition, this limits the applicability to several images and epidemiological studies. Therefore, the authors proposed an end-to-end methodology based on the CNN’s detection of location and disk abnormalities using two deep learning architectures. The first locates the papilla using DRIVE, Stare, DIRETDB1, and MESSIDOR databases. Additionally, the other identifies papillary abnormalities through a detector trained to classify the optic disc into three classes: normal, suspicious, and abnormal. The results had good quality in normal images, but there was a drop in performance under variable imaging conditions. The authors also used other databases, including the HAPIEE and PAMDI datasets. HAPIEE was collected from Lithuania (eastern Europe) and PAMDI from Italy (Europe). They used the CNN method and the Adaboost method with Haar-like features to segment glaucoma images. The results of these databases reported an accuracy of 86.5% (HAPIEE) and 97.8% (PAMPI).

The authors of [88] used the segmentation of blood vessels and optical disks using the VGG 19 model with slight modifications in the layers, named Deep Retinal Image Understanding (DRIU). The DRIU is a CNN capable of segmenting vessels and the optic disc with higher performance in terms of IoU and DICE for investigations of datasets than those obtained by human experts and used by [89] for results comparison.

In [90], the authors proposed using the automated segmentation of anatomical structures of background images, such as blood vessels and optical disks, based on the generative adversarial network (cGAN), which consists of two successive generations of networks: the generator and discriminator. The generator learns to map input characteristics from observation (retinal background color) to output (binary mask). The discriminator works with a loss function to train the process against the accurate discrimination of the image. The method was applied to two networks, DRISHTI-GS and RIM-ONE, and reached results of IoU (Jaccard) of 96%, DICE of 98%, and accuracy of 95.64%. The optical disc was segmented using fuzzy C-means clustering (FCM) in hospital samples and compared to standards.

Singh et al. [91] proposed a conditional generative adversarial network (cGAN) model to segment the optic disc. The cGAN is composed of a generator and a discriminator and can learn statistically invariant features such as the color and texture of an input image and segment the region of interest. The authors optimized a loss function that combines a conventional binary cross-entropy loss with an adversarial term that encourages the generator to produce output that cannot be distinguished from the ground truth. Skip connections were used for this method, concatenating the feature maps of a convolutional layer with those resulting from the corresponding deconvolutional layer. To train and evaluate the model, the DRISHTI-GS and RIM-ONE databases were used, reducing the size of the images to 256 × 256 and normalizing the value of each pixel to between 0 and 1. For optic disc segmentation, the model reaches values above 90% for accuracy, DICE and IoU for both databases.

In [92], deep neural networks are implemented for optic disc segmentation. Thus, the authors implemented faster R-CNN with different features extracted from the images available from the ORIGA dataset, reporting an accuracy of 91.3%.

The authors of [72] proposed a method of optical disk segmentation using the ResNet algorithm based on a CNN with a disc-aware and ensemble network (DENet). It compares the results with another five image databases, obtaining an AUC of 91.83% on SECS and 81.73% on SINDI.

The authors of [93] used a U-Net architecture to segment the optic disc and cup-based cGAN network consisting of two successive networks: the generator and discriminator. The proposal U-Net has fewer filters in all convolutional layers and does not possess an increasing number of filters for decreasing resolution. This model used three databases: DRIONS-DB, DRISHTI-GS, and RIM-One-r3. The images were then pre-processed with a contrast-limited adaptive histogram equalization and was then cropped by bounding boxes in the region of interest (ROI). The optic disc segmentation reached good results and the cup segmentation on both databases. The generator learned to map input characteristics from observation (retinal background color) to output (binary mask). The discriminator works with a loss function to train the process against the accurate discrimination of the image. The method reached IoU (Jaccard) results of 96% and DICE of 98%.

In [94], the authors proposed a retinal image synthesizer and a semi-supervised learning method for glaucoma assessment with deep convolutional generative adversarial networks (GANs). This allows the image synthesis and semi-supervised learning techniques, in which 86,926 publicly available images were used to create the model and evaluate them. The generated methods cropped retinal images for the identification of glaucoma. The implemented method reported an AUC of 90.17%, revealing its reliability.

The authors of [95] evaluated the perimetry with a combination of Laguna-ONhE and Cirrus-OCT, analyzing a total of 477 normal eyes, 235 confirmed, and 98 suspected glaucoma cases. The best results were verified with the combination of the “Globin Distribution Function” (GDF) and the threshold coefficient of variation (TCV). It was automatically analyzed with deep learning techniques, reporting an AUC of 99.5% (GDF) and 93.5% (TCV) with high sensitivity.

In [96], the authors intended to detect open-angle glaucoma with Bruch’s membrane opening (BMO)-based disc parameters, such as the BMO-minimum rim width (BMO-MRW) and the BMO-minimum rim area (BMO-MRA), in the Chinese population, in order to compare it with the retinal nerve fiber layer (RNFL) from optical coherence tomography (OCT) and the rim area (RA) from the Heidelberg retinal tomograph-III (HRT-III). The experiments were performed with 200 eyes of 77 healthy and 123 primary open-angle glaucoma (POAG) subjects analyzed with deep learning techniques and compared using the DeLong test. The reported results have an AUC of 94.6% (BMO-MRW) and 92.1% (BMO-MRA).

Several authors used different DL architectures and public and private databases with good results, as presented in Table 3. The location of the limits of the optic papilla is practical in pathologies that make it difficult to delimit the papilla, such as in myopic degeneration, myopic crescent, atrophic chorioretinitis, and sectorial papilla atrophy.

### 3.3. Segmentation of the Outer Limits of the Optic Disc and the Excavation by Detecting Glaucomatous Features of the Papilla

The segmentation of the outer limits combined with the excavation techniques allows the detection of the glaucomatous features of the papilla.

The authors of [97] implemented the Automatic Feature Learning for Glaucoma Detection Based on Deep Learning (ALADDIN), which uses a deep convolutional neural network (CNN) for the detection of glaucoma. The experiments were performed with the ORIGA and SCES datasets, reporting an AUC of 83.8% (ORIGA) and 89.8% (SCES).

The authors of [89] used a U-Net architecture for the segmentation of the optic disc and cup. The proposal U-Net has fewer filters in all convolutional layers and does not possess an increasing number of filters for decreasing resolution. This model used three databases: DRIONS-DB, DRISHTI-GS, and RIM-One-r3. The images were then pre-processed with a contrast-limited adaptive histogram equalization and then cropped by bounding boxes in the region of interest (ROI). The optic disc segmentation reached good results and cup segmentation on both databases. DICE reaches values above 80%, proving to be a reliable model.

The authors of [34] used a model based on the GoogleNet network for optical papilla detection and another for the detection of glaucoma. The results showed promising results with worst-quality images showing 90% accuracy in the HRF database, 94.2% in RIM-One-r1, 86.2% in RIM-One-r2, and 86.4% in RIM-One-r3.

In [98], the authors presented a U-Net-based convolutional neural network to trace corneal nerves to detect glaucoma. It is a fully automated framework which performs nerve segmentation with a sensitivity higher than 95% regarding manual tracing. It was revealed to be helpful for the detection of different features.

The authors of [99] implemented a deep learning system using fully convolutional neural networks (FCNs) to perform the segmentation of optic disk and cup regions. The authors analyzed the RIM-ONE dataset using one and two layers. The results reported an accuracy of 95.6% with one layer and 96.9% with two layers. The results reported 98% with one layer and 97.8% with two layers regarding the AUC.

In [100], the authors used the Transfer Learning, GoogleNet, and Inception-V3 techniques with multi-modal data from 1542 retinal fundus images for the glaucoma diagnosis. The results reported an accuracy of 84.5% and an AUC of 93%.

In [101], the authors used 1542 photos (with different sizes and cropped to the size of 240 × 240 pixels) using a Nidek AFC-330 non-mydriatic camera, divided into groups of 754 for training, 324 for validation, and 464 for tests—among which 786 were normal, 467 showed advanced glaucoma; and 289 showed early glaucoma. The authors classified them through CNN using TensorFlow, and the same datasets were used to pre-trained GoogleNet models. Inception-V3 reported 82.9% accuracy for training, 79.9% for validation, and 77.2% for test. Transfer Learning, GoogleNet, and Inception-V3 then achieved 99.7% accuracy with training data, 87.7% with validation data, and 84.5% with test data. Finally, they concluded that both early and advanced glaucoma could be correctly detected by machine learning using only background photos. Nonetheless, the model has shown greater efficiency in detecting early glaucoma than previously published models and argues that Transfer Learning is an attractive option in building a classification model of images.

The authors of [102] analyzed 48,116 background photographs by trained ophthalmologists using a deep learning algorithm. Glaucomatous papillae were defined with a vertical diameter > 0.7 and other typical changes. The deep learning system detected glaucomatous papillae with a high sensitivity of 95.6% and a specificity of 92.0%. It also reported an AUC of 98.6% with 87 false negatives.

In [32], the authors performed glaucoma diagnosis with a CNN with eighteen layers. It extracted robust features from 1426 fundus images, among which 589 were normal and 837 showed glaucoma. The methods reported an accuracy of 98.13%, a sensitivity of 98%, and a specificity of 98.3%.

The authors of [103] proposed a method with DenseNet incorporated with an FCN with a U-shaped architecture that is a CNN with nineteen layers. This deep network encourages feature re-use and reduces the number of parameters to improve the optic disc and cup segmentation. The approach of Al-Bander used five databases of color fundus images: ORIGA, DRIONS-DB, DRISHTI-GS, ONHSD, and RIM-ONE. For the pre-process, only the green channel of the color images was considered since the other color channels contain less helpful information. The images were then cropped in the ROI, and to artificially increase the number of images, augmentation processes with vertical flips and random crops were performed. For optic disc segmentation, the model reached better results in DICE and IoU for the DRISHTI-GS database than RIM-ONE, and the cup segmentation confirmed the same thing. DICE and IoU still obtained lower values compared to the optic disc segmentation. The system was first trained and tested with the same database (ORIGA) and then tried in other databases, reaching a DICE of 87.23%, a Jaccard score of 77.88%, an accuracy of 99.86%, a sensitivity of 87.68%, and a specificity of 99.94% for optic papilla; and a DICE of 96.4%, a Jaccard score of 93.11%, an accuracy of 99.89%, the sensitivity of 96.96%, and the specificity of 99.94% for excavation, respectively.

The authors of [104] used CNN with U-Net architecture and a reduced number of filters in each convolution to perform segmentation in the DRIONS-DB, RIM-ONEv3, and DRISHTI-GS databases with results (IoU of 89% and DICE of 94% in DRIONS-DB and 95% in RIM-One-r3) comparable to other state-of-the-art methods including Maninis’ DRIU and Zilly’s BCF. In addition, the technique showed reliable segmentation quality and applicability in image identification tasks.

In [105], the authors developed a hierarchical deep learning system (HDLS) using 1791 fundus photographs for glaucoma diagnosis. Its recognition accuracy was 53% for the optic cup, 12% for the optic disc, and 16% for retinal nerve fiber layer defects. The authors needed to test the methods with a significant sample rather than an extremely small dataset.

The authors of [106] used a deep neural network to detect glaucoma and calculate the vertical cup-disc ratio. They used the UZL test set of 2643 images, reporting an AUC of 94% for glaucoma detection.

In [107], the diagnosis and localization of glaucoma were performed with the acquisition of fundus, retinal nerve fiber layer (RNFL), optical coherence tomography (OCT) disc, OCT macula, perimetry, and perimetry deviation images. The implemented methods were convolutional neural networks (CNNs) and gradient-weighted class activation mapping (Grad-CAM) with a large dataset from the Samsung Medical Center (SMC). The results reported an accuracy of 96%, a sensitivity of 96%, and a specificity of 100% for optic disc images.

Several automated DL methods have been presented that can help locate the outer limits of the papilla and excavation and directly classify the existence or not of glaucoma, as shown in Table 4.

Thus, automated methods using DL architectures have obtained good results in tracking and classifying glaucomatous papilla and can help the specialist organize glaucoma through the characteristics in the images. Nonetheless, few studies have included clinical records or even results of subsidiary exams in the networks.

## 4. Segmentation Methods

Optical disc and cup segmentation use processes that help locate and evaluate the papilla through its metric characteristics determined by nuances of color, texture, optical disc delimitation, and cupping [108]. At first, in the ophthalmological routine, the specialist qualitatively calculates it through the direct observation of the papilla to compare the two eyes. The segmentation of the excavation is more complex in the optic disc due to the nerve fiber layer [89]. This causes imprecision in the excavation limits which is impaired by the presence of blood vessels and conditions that result in the alteration of the limits of the papilla and the excavation as atrophy, drusen, or edema hemorrhage [109].

Whether manual or automated, segmentation methodologies have limitations and low image resolution, media opacity, and photographic artifacts, such as lighting problems and image distortion [110]. Pathological cases with manifestations at the limits of the optic disc, such as papillary atrophy, influence the precision of segmentation [109]. The segmentation of the excavation is hampered by the presence of blood vessels that cover part of the excavation, and the variation in the intensity of the edge color of the hole makes it challenging to delineate the excavation limits, making the detection of the excavation’s external boundaries a challenging task [111]. Bock et al. used methodologies inspired by facial and object recognition which do not require papilla segmentation and obtained 75% sensitivity and 85% reproducibility in detecting glaucomatous papillae [112]. However, this depends on more expensive cameras and machinery.

Furthermore, this requires many positive or negative glaucoma samples for screening. The recognition of papilla is more susceptible to errors because of the subtlety of its appearance [113]. These factors make it challenging to recognize the papilla by a method only based on appearance [114]. However, in the future, it is possible that, with the advancement of technology for obtaining high-resolution images, glaucoma can only be detected by appearance [115].

The metrics for the automatic segmentation methods of the main structures of the optic nerve head formed by the optic disc and the cupping are a process which can aid in tracking, identifying, and evaluating the progression of glaucomatous disease [116]. However, this is a complex process which requires consideration of the subtlety and variability of the anatomy of the nerve fiber layer, irregular contours due to glaucomatous damage to nerve fiber [117]. In addition, the presence of blood vessels can impair the excavation delineation or visualize the limits and excavation of the papilla by deflecting the regions of atrophy of the fiber layer [118].

Manual or automated methods have limitations, and the low image resolution and anatomical noises constitute a rule [110]. In addition, fixing the exact limits that characterize the normal glaucomatous papilla becomes a constant challenge due to local anatomical variability, both under normal conditions and in the face of some pathologies that make it difficult to establish the exact limits of the excavation [118]. Finally, optical disc segmentation requires a pre-processing step, including image channel selection, illumination normalization, contrast enhancement, and the extraction of blood vessels [111].

The authors of [119] didactically grouped the segmentation methods into five large groups. The superpixel technique includes many studies for segmenting the optic disc regions and excavation, followed by clustering techniques, mathematical morphology, an active contour, and a convolutional neural network. However, there is no consensus on the best approach [120]. The different groups are as follows.

1. **Clustering algorithms:** Segmentation is performed pixel by pixel, using information from readings through RGB and HSV color channels. These have the advantages of simplicity in terms of implementation and low requirements in terms of computational time; and, as disadvantages, problems in defining the best set of attributes, sensitivity to noise, initialization of centroids and which group represents each region. For example, the authors of [121] obtained an excavation F-Score of 97.50% in 59 images from the local Ophthalmological Hospital, DIARETDBo, and RIM-One-r1, whilst the authors of [122] obtained an excavation accuracy of 97.04%, evaluating the CDR in 209 images from the DRIHTI-GS and RIM-One-r3 databases. Among the main clustering algorithms are:
  - *K-Means:* Unsupervised algorithm that divides images into parts based on a model created by averaging each piece. Its disadvantage is its sensitivity to inconsistent values, noise, and initial centroids;
  - *Fuzzy K-Means:* Unsupervised algorithm often used in medical images based on the mean of each group which groups similar data values using fuzzy logic to calculate this similarity. It has the advantage of being efficient in the segmentation of images with noise.
2. **Superpixel:** Based on partitioning the image into multiple pixel clusters and analyzing the image to be examined by regions, it has the advantage of less interference from image noise and the disadvantage of a pre-processing step with the risk of data loss related to the image edge. The best results were obtained by [123] using CDR and ISNT evaluation metrics in 101 DRISHT images with a cupping accuracy of 98.42% and an optic disc accuracy of 97.23%;
3. **Active contour:** The detection and imaging using curved evolution techniques can represent the curve as it allows a topology change. The disadvantage is that any change in the initial curve and the object to be detected modifies the result, making the method extremely sensitive to initialization. The best results were obtained by [124], who obtained an accuracy of 99.22% with the advantage of allowing the segmentation of the optic disc regions and the cup using low-quality images;
4. **Mathematical morphology:** The image is improved through morphological operations, including dilation, erosion, opening, and closing. It has the advantage of the simplicity of its implementation and the disadvantage of choosing the right structural element to transform intellectual intuition into practical application. The authors of [125] obtained excellent results in detecting glaucoma with 96% correct answers in the CDR and ISNT ratios.
5. **Convolutional neural network:** The use of neural networks to recognize and classify images and videos requires less pre-processing to homogenize optical disc images in terms of image quality, brightness, and contrast. Moreover, the same network can recognize patterns with different photos of different objects compared to other methods. For example, the authors of [126] obtained an F-score of 83.5% for the excavation, 94.5% for the optic disc, 72% for the excavation overlay, and 89% for the optic disk overlay—evaluating 319 images with F-Score evaluation metrics and overlay on DRIONS-DB, DRISHTI-GS, and RIM-ONEv3. In [127], it was verified that convolutional neural networks have been gaining ground and proving to be a powerful tool for segmentation, emphasizing that a large set of images is needed to train these networks.

Different algorithms offer good results in detecting and segmenting the optic disc and cupping. Nonetheless, many have limitations due to the use of images with varying sharpness along the edge of the optical disc and cupping, variability in the structure of the optic papilla in normal eyes, of the presence of peripapillary atrophy, of the papillary drusen—structures that alter the limits of the optical disc, which may cover it entirely in more advanced cases—and of the path of blood vessels whose deflection plays an essential role in delimiting the limits of the excavation—which by another side may mask and make it difficult to delimit the inner border of the papilla.

Retinal pathological images that evolve with changes in the optic nerve head should be considered to obtain correct CDR and ISNT calculations for glaucoma screening, including sectoral and diffuse papillary atrophy, peripapillary atrophy, papilla insertion changes, and papilla drusen. According to [128], most current methods were tested on a limited number of datasets, such as DRIVE and STARE. These datasets do not provide images with many different characteristics. Furthermore, the generally low resolution of the images (ranging from 0.4 to 0.3 megapixels) made the segmentation process even more challenging. Most retinal images used to assess segmentation methods were taken from adults, and it was not always possible to compare the two eyes. The retinas of babies have different morphological characteristics to those of adults, and this difference must be considered in segmentation methodologies.

Chakravarty et al. [129] proposed joining disc segmentation, optical cupping, and glaucoma prediction by dividing CNN characteristics into different tasks to ensure better learning. The segmentation masks were placed on separate channels, and the CNN and encoder outputs were combined and fed to a single neuron to predict glaucoma.

In [130], the authors used one of the first deep learning architecture models for papilla segmentation to calculate cupping and the presence of glaucoma to overcome the need for artisanal methods of papilla segmentation. Since then, there have been significant advances in the architectures used in neural networks.

The authors of [131] reported some disadvantages of [103] when using grayscale images and dropout layers at different stages that resulted in data loss and proposed batch normalization on CNN for optical disk segmentation.

In [132], the authors used the U-Net in neural networks to segment the optic disc and excavation in images from the REFUGE database, divided into two training and validation groups with 400 pictures each. A backpropagation function generated a segmented image closer to the true one. Two successive networks were used: the first network works as a generator network used to segment the input images; and the second network works as a simple CNN network used to extract predicted features. The method distinguished between the disc and cup regions with an accuracy of 93.40% and 83.42% and MAE CDR 6.05%.

The authors of [133] proposed a deep learning model with an architecture called a fully convolutional network (FCN) and another one called dilated residual inception (DRI) to estimate the depth of the monocular excavation. For ORIGA, the authors obtained an AUC of 81.39% and an AUC of 85.08% for the ResUnet network.

In [31], the authors used a multi-branch neural network (MB-NN) model to extract the areas of images relevant to measuring different features. The model included a Faster-R-CNN method in a dataset of 2000 images. The technique reported an accuracy of 91.51%, a sensitivity of 92.33%, and a specificity of 90.90%.

The authors of [134] selected the VGG19, GoogleNet (also known as Inception-V1), ResNet50, and DENet models for the automatic classification of Glaucoma. Valverde compared the performance of Transfer Learning and training from scratch with this model. To confirm the performance of VGG19, 10-fold cross-validation (CV) was applied. Valverde used 2313 retinal images from three different databases: RIM-ONE, DRISHTI-GS (public), and Esperanza (private dataset). In the RIM-ONE database, the images classified as suspect were considered for the study as glaucomatous. The photos did not suffer any correction or modification of illumination or contrast enhancement; they were simply processed to a common and standard format to train the networks homogeneously. The best result was obtained with the VGG19 model using Transfer Learning.

In [135], the authors analyzed fundus photographs for retinal blood vessel segmentation using contrast-limited adaptive histogram equalization (CLAHE) with local property-based intensity transformation (LPBIT) and K-means clusters. They used four datasets, including Structured Analysis of the Retina (STARE); Digital Retinal Images for Vessel Extraction (DRIVE); CLAHE; and LPBIT. They implemented the K-means clustering (KNN), reporting accuracy of 95.47% in the segmentation of glaucoma.

The authors of [135] proposed a method for automatically diagnosing an optic disc that is segmented with intensity thresholding and morphological operations. They used the ORIGA, RIM-ONE-r3, DRISHTI-GS, Messidor, DRIONS-DB, and DIARETDB1 to apply intensity thresholding and morphological operations optic disc segmentation. The results reported 98.75% accuracy in the segmentation of glaucoma.

In [136], the fundus photographs were analyzed with a computer-aided diagnosis (CAD) pipeline capable of diagnosing glaucoma with mobile devices. They used different datasets, including ORIGA, DRISHTI-GS dataset, iChallenge, RIM-ONE, Retinal Fundus Images for Glaucoma Analysis (RIGA), and other methods including CNN, MobileNetV2, VGG16 and VGG19, Inception-V3, and ResNet50. Finally, it reported 90% of accuracy in glaucoma recognition.

The authors of [137] executed experiments for automated glaucoma diagnosis and the segmentation of the optic disc and optic cup with different datasets, including G1020 and ORIGA. The implemented methods were the region-based convolutional neural network (R-CNN), Restnet50, and Inception-V3, reporting an F1-score of 88.6%.

In [138], the authors used a threshold-based algorithm to segment the optic disc and a modified region growing algorithm for optic cup segmentation. The segmentations were followed by infilling blood vessels and morphological operations. The datasets used were DRISHTI-GS, reporting a DICE score of 94% with SVM in glaucoma classification.

The authors of [139] used the cup-disc encoder-decoder network (CDED-Net) architecture with dense connections for the joint segmentation of the optic disc and optic cup. For the model training, the authors used the DRISHTI-GS, RIM-ONE, and REFUGE datasets, and the model includes the SegNet (VGG16) method, which reports 95.97% accuracy.

## 5. Classification Methods

Sun et al. [140] used the Inception-V3 architecture to detect glaucomatous optic neuropathy. The researchers underwent image analysis by specialists in ophthalmology before applying the algorithms. Color subtraction techniques were applied during pre-processing to even out the varied lighting.

The authors of [141] developed an algorithm for classifying glaucomatous papillae with great sensitivity and comparable specificity and proved the algorithm’s effectiveness in front of specialists. The algorithm’s performance reported an AUC of 0.945 for glaucoma detection using a reference standard from the glaucoma experts, and 0.855 using a reference standard from other ocular care providers.

In [142], the authors proposed a DL method to screen glaucoma in retinal fundus images using a database granted by the Singapore National Diabetic Retinopathy Screening Program with several eye diseases. The DL system recognizes the characteristic of referable diabetic retinopathy, possible glaucoma, and AMD, and showed the results that can be used to screen glaucoma.

In [143], the ResNet50 and GoogleNet models were selected, training them with two public databases: a database from Kim’s Eye Hospital (a total of 1542 images, including 786 photos from normal patients and 756 from glaucoma patients) and RIM-One-r3. All fundus images were histogram equalized, and the database from Kim’s Eye Hospital was used to train the two models. For the performance evaluation, the models were tested with the RIM-One-r3 database. GoogleNet obtained better results for early stage glaucoma than for the advanced-stage glaucoma.

The authors of [144] used a DL network with Transfer Learning with the weights of ImageNet. The two DL models used were VGG19 and Inception ResNet V2. These two models were pre-trained and then fine-tuned. For this work, two databases were used: one from the University of California Los Angeles (UCLA) and the other, publicly available, called high-resolution fundus (HRF). The authors randomly selected 70% of the images the UCLA database for training, 25% for validation, and the remaining 5% for testing. The models were then re-tested with the HRF database to bolster work. The Inception ResNet V2 model for the UCLA database obtained a specificity and sensitivity above 90% even when re-tested with the HRF database.

In [68], the authors applied five different ImageNet-trained models (VGG16, VGG19, Inception-V3, ResNet50, and Xception) for glaucoma classification using a ten-fold cross-validation strategy to validate the results. These models were fine-tuned, and the last fully connected layer of each CNN was changed for a global average pooling layer followed by a fully connected layer of two nodes representing two classes with a SoftMax classifier. Diaz did two experiments which varied the number of fine-tuned layers and epochs. This work collected five databases: ACRIMA, HRF, DRISHTI-GS, RIM-ONE, and Sjchoi86-HRF. The images were cropped around the optic disc using a bounding box of 1.5 times the optic disc radius. The photos were augmented using random rotations, zooming in a range between 0 and 0.2, and horizontal and vertical flipping to avoid overfitting. All the models passed 96% of AUC—which is an excellent result.

In [145], a CNN architecture was introduced based on boosting which shared some of the characteristics of ensemble learning systems. An entropy sampling method was shown to obtain results superior to those of the uniform sampling. The proposed method was a practical approach to learning convolutional filters without large extensive data required for training. Instead of backpropagation, each stage of the filters is learned sequentially using boosting. Each step considers the final classification error to update itself and not the backpropagated error, and instead of image-level data, this method operates on patch-level data. The RIM-One-r3, DRISHTI-GS, and Messidor databases were used to train the models. First, the optic disc is localized with a circular Hough transform on the green channel in each database. The image is cropped so that the optic disc is central in the picture. Then, the image is converted from RGB to L * a * b color space using a nonlinear transformation that mimics the nonlinear perspective response of the eye. The intensities are then normalized between 0 and 1. For the optic disc and the cup segmentation, the DRISHTI-GS achieved better results in terms of DICE and Intersection-over-Union (IoU) than RIM-One-r3.

The authors of [132] proposed neural network constructs utilizing the FCN and the inception building blocks in GoogleNet. The FCN is the main body of this method’s deep neural network architecture. They added several convolution kernels for feature extraction after deconvolution, based on the Inception structure in GoogleNet. The authors’ experiments used two databases: REFUGE and one from the Second Affiliated Hospital of the Zhejiang University School of Medicine. This technique used a fully automatic method—namely the Hough Circle Transform—which recognizes and cuts the image to obtain a picture of the ROI. The image data are increased by rotating, flipping, and adjusting the contrast, before using the Laplacian for image enhancement. Since the red channel contains less helpful information, this paper only uses blue-green media. In the optic disc and cup segmentation, the model reached values above 90% for the DICE and the IoU.

In [108], a modified U-Net was developed with a pre-trained ResNet-34. This work was composed of two steps: first, one single label-modified U-Net model applied to segment an ROI around the optic disc, followed by a cropped image used in a multi-label model with the objective of simultaneously segmenting the optic disc and cup. In the study of Yu, the RIGA database was used to train and evaluate the CNN but then, to achieve robust performance, the model trained on RIGA was applied on the DRISHTI-GS and RIM-One-r3 database. These image databases were pre-processed with a contrast enhancement and resized to 512 × 512 dimensions. Data augmentation was applied with rotation tricks, upside-down flip, and a left-right flip. Since the segmentation was considered a pixel-level classification problem, the binary cross-entropy logistic loss function was used. In this method, the segmentation of the optic disc and the cup reached better results with DRISHTI-GS than with RIM-One-r3.

In [146], an automated method is proposed for identifying retinal fiber layer defects (RNFLDs) using a classifier called a recurrent neural network (RNN), previously trained in 5200 regions with 13 training images. The proposed method successfully detected 14 out of 16 RNFLD bands and 1 false negative with an accuracy of 87.5%.

The authors of [147] used a backpropagation neural network to classify the retinal nerve fiber layer (RNFL). This uses 40 background images as a test and 160 sub-images (80 with normal RNFL and 80 with diminished RNFL). This resulted in 94.52% accuracy due to high myopia (42.6%), diabetic retinopathy (4.6%), DMS (3.4%), and false positives due to increased physiological cupping.

In [148], the authors used a gradient-based classification activation map (Grad-CAM), applied attention mining (AM) based on these Grad-CAM results, and performed dissimilarity (DISSIM) loss for training. They used a private dataset from 13 universities, and complementarily, they used deep convolutional neural networks (DCNNs) and the VGG19 model to obtain the recognition of glaucoma with an accuracy of 96.2%.

The authors of [149] evaluated a smartphone application-based deep learning system (DLS) for detecting glaucomatous visual field changes named iGlaucoma. The mobile application executes CNN and a modified ResNet-18 to classify glaucoma. They used a private dataset, which reported an AUC of 87.3%.

In [150], the authors used OCT images with two deep learning networks for scleral spur localization and angle-closure classification. The dataset used was the Angle-Closure Glaucoma Evaluation Challenge (AGE), in which the deep convolutional neural network (DCNN) and ResNet18 methods were applied for reporting with 100% accuracy.

The authors of [151] used the Ocular Hypertension Treatment Study (OHTS) dataset for the implementation of deep archetypal analysis (DAA) for feature extraction and the class-balanced bagging for classification of glaucoma diseases. The results reported an AUC of 71%.

The choice of automated methodology was based not only on the results obtained in the networks but also on clinical data being the closest form to the methods used by specialists. However, when adding data from the clinical history, there was no significant difference in the AUC curve. Nonetheless, this increased the sensitivity and specificity values, indicating that this information can improve classification values [134].

## 6. Discussion

After reconstruction, the quality of photographic images may be improved by adding certain features through deep learning. For example, in the “active acquisition” described by [152], multiple photos of the same structure will be automatically reconstructed by a learning algorithm, resulting in the best quality image and emphasizing vital diagnostic features—for example, MRI and 3D tomography images. In ophthalmology, it is necessary to define the “loss of function” to minimize the error in automatic reconstruction. However, image restoration and classification processes have not been applied simultaneously, and no authors have used real-time image reconstruction.

Some traditional image processing algorithms can be used in a deep learning structure for image restoration, such as the BM3D80 algorithm used by [153]. This has been shown to outperform many noise removal networks that have been replaced by actual photographic noise. However, this requires the creation of a multi-modality multi-frame database from multiple manufacturers for a realistic assessment of general image restoration networks.

Deep learning techniques have been proven capable of solving several image artifacts caused by movements of acquired images (a common source of blurring of retinal images), static blurs, mirroring, and aberrations caused by ocular means.

Eyes with pre-perimeter open-angle glaucoma (OAG) had a better diagnostic performance using deep learning rather than machine learning techniques.

Optical disc characteristics in background images to reduce the influence of optic disc misalignment for diagnosing glaucoma had an AUC of 83.84%, a value which is very close to the results of manual detection.

Existing CNN architectures used in the medical image recognition field include AlexNet, VGG, ResNet, and GoogleNet. The AUC curve is the most used evaluation metric to assess a diagnostic model of AI. It varies from 50% to 100%, and the higher the model’s performance, the better it is. Sensitivity and false-positive values are compared.

Despite the excellent performance of the DL, it has several limitations that limit its use in practical applications, namely:

- The need for continuous learning with the help of systems so that models can improve;
- Potential forgetfulness when updating models;
- The high dependence on data quality, as different image services containing other noises can affect different image protocols and influence models and performance;
- Incorrect results arising from learning the network with multi-referential training data, that is, with biased characteristics pointed out by several experts;
- Possibility of adding other factors such as visual acuity, refractometry, presence of familial glaucoma, ocular history (e.g., genetic and degenerative diseases of the anterior segment, cataracts, and choroidal diseases), and systemic factors (e.g., glycemic control, and diabetic vascular diseases), and other comorbidities that current algorithms may not incorporate, the severity of the illness, and the urgency of the referral;
- Particular (individual) image-processing techniques are required according to the severity of the disease;
- Errors inherent in training networks with only one type of image, for example, images with a slightly temporal optic disc, cause the network to incorrectly learn to associate the temporal location of the disc with the presence of the disease;
- Existing datasets are still insufficient and should contain a more significant number of images with normal anatomical variations of the papillary region;
- Population characteristics and phenotypes should be considered when input data are selected. DL architectures are based on training data from different databases. There is a lack of more robust studies that consider individual clinical particularities to classify into disease and non-disease, in addition to these databases requiring permanent data updating.

A single abnormality detected using an imaging technique cannot always guarantee the correct diagnosis of retinal diseases such as glaucoma.

Increasing the number of images in the database and the number of manual segmentations by experts from different countries can increase the robustness of future results [97].

Recent works have confirmed that machine learning has shown promise for aiding in the diagnosis of glaucoma and as a future instrument for monitoring the disease, with greater population inclusion.

Additionally, recent literature reviews have not emphasized the details of the functioning of the different deep learning algorithms used in CADs for ophthalmological diagnoses through optical papilla images [132]. Although they have not offered robust criteria to discuss the advantages and disadvantages of different algorithms, CNNs have been proven to be a valuable alternative in the automated classification of glaucomatous papillae. However, more powerful tests of CNN architectures trained in other databases and papilla images are needed to better establish the reproducibility of the methods.

Other studies have shown reliable results in identifying and performing the automatic segmentation of optical disc structures through machine learning using only background photos. In addition, this can differentiate normal and glaucomatous papillae as well as classify different stages of early (Gi), moderate (Gm), and severe glaucoma (Gse).

The authors reached reasonable accuracy, sensitivity, and specificity levels when using neural network models with different architectures and algorithms to classify normal and glaucomatous papillae.

The RIM-ONE, DRISHTI-GS, and DRIONS-DB databases were widely used as image standards, and U-Net with FCN architecture was the algorithm that seemed to offer better performance. However, other studies will be essential to increase the credibility of the methods used against the subtleties of papilla structures [93] and reduce the resistance of professionals to the use and reliability of the automated procedure.

Among the different classification methods proposed, most were based on the extraction of anatomical features and a small part on image textures. Perhaps combining these techniques could result in the ideal recognition of glaucoma. Furthermore, unlike past subjective assessments, ophthalmic imaging provides objective, accurate, reproducible, and quantitative data that can be tracked with statistics [154].

The automatic tracking of glaucomatous papilla through photographs has revolutionized the diagnosis of glaucoma. Furthermore, it has been proven to be an essential auxiliary diagnostic tool due to the subtleties resulting from the anatomical variants in the papilla “in vivo”, capable of obscuring the excavation limits.

Although ophthalmic diagnoses are feasible and effective, none of the authors have dealt with detailed reviews of different state-of-the-art deep learning algorithms used in retinal imaging (including glaucoma) ophthalmological diagnoses. The authors of [132] pointed out that the most used architectures in studies of the diagnosis with background images were the FCN, ResNet, AE, and lists the following limitations:

1. The lack of availability of large datasets is a problem because the model learns from large amounts of data. The model proposed by [155] may be an essential solution to this problem, but little effort has been made to synthesize new annotated background images and adequate clinical relevance. The generative contradictory network of automatic variational encoders is a trendy architecture for imaging. Their application can generate large amounts of clinically relevant synthetic data that will help increase the amount of data and prevent issues of data privacy.
2. Due to differences in camera configurations, in most literature, training data come from the same image distribution, which does not occur in real life. Transfer learning has been used for different applications in this area as well as subdomain adaptation (a subdomain of transfer learning), where data for training and testing are extracted from other distributions. However, it is not always possible to obtain data from training and testing the same distribution in the real world. Therefore, the model must be robust to test data from a different distribution. Accuracy often decreases due to this domain-shifting problem, and more emphasis should be placed on in-depth domain adaptation approaches to create robust models that can be implemented for real-world ophthalmological diagnosis.

Aspects related to clinical history such as age, race, eye trauma, family history, eye pressure, in addition to anamnesis characteristics such as cataract, eye pressure, the presence of artifacts in the anterior chamber, drainage angle (Schlen), and corneal thickness proposed by [31] could contribute to greater diagnostic accuracy and individualized glaucoma.

The new technologies of portable cameras combine the increase in quality, the potential for using images, and the ease of incorporation with smartphones, reduce costs, increase portability and the minimum requirement for learning by the examiner, produce a more accurate diagnosis, and can lead to earlier treatment [156].

In the future, if integrated into primary care, automated systems may reduce or eliminate unnecessary referrals and enable ophthalmic self-monitoring by patients through smartphone retina photography, visual acuity, and visual field tests which would facilitate referral to specialists, diagnosis, and the treatment of eye diseases.

Other future applications of deep learning in the eye clinic include patient self-examination, the obtention of photos by a technician in a virtual clinic or a hospital waiting room before the eye appointment. In addition, patients could be scanned in remote areas by a healthcare practitioner via home monitoring to assess disease progression.

Health systems suffering from labor shortages can benefit from modern automated imaging. With the increase in AI, the role of the physician will evolve from the paternalism of the 19th century and evidence-based medicine of the 20th century towards a more individualized clinical work focused on the patient, with the improvement of data quality, taking advantage of previous already structured clinical experiences in data/evidence.

Despite the development of deep learning in ophthalmology, few prospective clinical trials have evaluated its performance in real and everyday situations. The IDx-DR was recently approved as the first fully autonomous diagnostic system for diabetic retinopathy, but the patient benefit in terms of visual outcome remains unclear.

Glaucoma screening is performed in the eye exam and depends on several racial factors, age, family, the use of some medications, and ocular factors such as nearsighted or farsighted eyes. In addition, eye pressure represents a risk factor. However, the characteristics of the papilla mean a significant diagnostic factor and are visible in the intermediate and advanced stages of the disease. With artificial intelligence assessment solutions, the diagnosis of glaucoma is solely based on the appearance of the optic papilla without considering others risk factors. Therefore, they can falsify the tracking data.

On the other hand, different studies show reliable results in the identification of glaucoma using specific characteristics of the glaucomatous papilla and data from the anamnesis. These factors bring the technology closer to the diagnostic reality. However, as this is a multifactorial diagnosis, greater individualized data on that patient may obtain better results. Ultimately, with information that does not provide individual answers for each patient, one risks treating everyone identically.

Regarding patient management, these numbers will not reassure patients likely to develop or already suffering from the disease but face a waiting period for diagnostic confirmation or treatment initiation, even in results that point to a safe glaucoma screening through AI. In medicine, any treatment is justified when the benefits are assumed to outweigh the disadvantages. It has been proven that AI can help track glaucoma in a papillary appearance. However, establishing a definitive diagnosis and monitoring treatment depends on other parameters. The decision to start treatment is simple in cases in which there is evidence to confirm the characteristics of glaucoma either by specialists or through AI, especially in moderate and advanced topics.

Regarding the problem of starting the treatment to reduce the influence of the risk factors (e.g., intraocular pressure), the decision can be very delicate. It is vital when an immediate danger does not threaten the patient’s vision, nor the certainty that the risk factor is not is decisive for visual deterioration. Thus, it can lead to the imposition of preventive treatment, even if the hypothetical advantage is very modest.

## 7. Conclusions

This literature review showed that the analysis of images of the papilla through deep learning methods allows for greater precision in tracking the glaucomatous papilla through optical papilla images. In addition, the insertion of clinical data promoted a significant difference in the AUC curve, corroborating the results obtained by the authors of [134]. Nonetheless, it may have clinical importance in the individualized diagnoses of suspected glaucoma cases.

Methodologies that use public image banks for glaucoma screening need a constant increase in the availability of data that can encompass the normal and pathological morphological diversities of the fundus.

The literature has shown that screening for glaucomatous papillae by deep learning techniques can improve diagnostic accuracy and help screen for glaucoma through the classification of papillary images. However, this still demonstrates weaknesses considering the small amount of data from public databases in the sampling of images obtained by different cameras and the association of data obtained by various architectures.

In the future, we hope to obtain better quality images with the technical improvement of the Internet, smartphones, and applications that can help specialists track glaucoma, especially in more distant places. We may popularize, cheapen, and improve the accuracy of the recognition of papilla glaucomatous, especially in its early stages. Developing a reliable solution is the key to promoting acceptance of these methods among patients and doctors and promote patient empowerment.

## Figures and Tables

**Table 1:** Summary of the characteristics of the public databases.

Database	Glaucoma/Normal	Optic Disc/Cup	Total
ACRIMA	396/309	No	705
DRIONS-DB	-	No	110
DRISHTI-GS	70/31	Both	101
HRF	27/18	Both	45
ONHSD	-	Optic disc only	99
ORIGA	168/482	No	650
REFUGE	40/360	Both	400
RIM-One-r1	194/261	Both	455
RIM-One-r3	74/85	Both	159
Sjchoi86-HRF	101/300	No	601

**Table 2:** DL methods for glaucoma screening.

Study	Methods	Databases	Results
[75]	DNN	ORIGA	AUC: 0.94
		RIM-One-r3	
		DRISHTI-GS	
[76]	DeepLabv3+	RIM-ONE	Accuracy: 97.37% (RIM-ONE), 90.00% (ORIGA), 86.84% (DRISHTI-GS) and 99.53% (ACRIMA)
		ORIGA	
	MobileNet	DRISHTI-GS	AUC: 100% (RIM-ONE), 92.06% (ORIGA), 91.67% (DRISHTI-GS), and 99.98% (ACRIMA)
		ACRIMA	
[77]	U-Net	DRISHTI-GS	AUC: 94%
		REFUGE	
		RIM-One-r3	
[78]	MFPPNet	Direct-CSU	AUC: 90.5%
		ORIGA	
[79]	Fuzzy broad learning	RIM-One-r3	AUC: 90.6% (RIM-One-r3) and 92.3% (SCRID)
		SCRID	
[80]	U-Net	N/D	DICE: 89.6%
			Precision: 95.12%
[81]	DNN	5716 images	AUC: 94%
[82]	DNN	933 healthy and 754 glaucoma images	Sensitivity: 73%
			Specificity: 83%
[83]	M-Net	REFUGE	DICE: 94.26% (optic disc) and 85.65% (optic cup)
			AUC: 96.37%
			Sensitivity: 90%
[84]	DL-ML Hybrid Model	HRF	Accuracy: 100%
			Sensitivity: 100%
[85]	GlaucoNet	DRISHTI-GS	Overlapping score:
		RIM-ONE	- Optic disc segmentation: 91.06% (DRISHTI-GS), 89.72% (RIM-ONE), and 88.35% (ORIGA)
		ORIGA	- Optic cup segmentation: 82.29% (DRISHTI-GS), 74.01% (RIM-ONE), and 81.06% (ORIGA)
[86]	CNN	282 images with 70 glaucoma cases and 212 normal cases	Sensitivity: 95.48%

**Table 3:** Classification of glaucoma by DL methods for the segmentation of the outer limits of the optic disc.

Study	Architecture	Databases	Results
[87]	CNN	HAPIEE	Accuracy: 86.5% (HAPIEE), and 97.8% (PAMPI)
		PAMDI	
[88]	CNN	DRIONS-DB	Accuracy: 97.1% (DRIONS-DB) and 95.9% (RIM-ONE)
		RIM-ONE	
[90]	CNN	N/A	Accuracy: 95.6%
[91]	CNN	N/A	Accuracy: 92.7%
[92]	Faster R-CNN	ORIGA	Accuracy: 93.1%
[72]	ResNet	SCES	AUC: 91.8% (SECS) and 81.8% (SINDI)
		SINDI	
[93]	U-Net	DRIONS-DB DRISHTI-GS RIM-ONE	IoU: 96.0%
			DICE: 98.0%
[94]	GANs	86926 images	AUC: 90.17%
[95]	DNN	477 normal eyes, 235 confirmed, and 98 suspected glaucoma cases	AUC: 99.5% (GDF) and 93.5% (TCV)
[96]	DNN	200 eyes of 77 healthy and 123 primary open-angle glaucoma	AUC: 94.6% (BMO-MRW) and 92.1% (BMO-MRA)

**Table 4:** Classification of glaucoma by DL methods for segmenting the outer limits of the optic disc and cup.

Study	Architecture	Databases	Results
[97]	CNN with six layers	ORIGA	AUC: 83.1% (ORIGA) and 88.7% (SCES)
		SCES	
[89]	U-Net	DRIONS-DB	IoU: 89% (DRIONS-DB), 89% (RIM-ONE-r3), and 75% (DRISHT-GS1) DICE: 94% (DRIONS-DB), 95% (RIM-ONE-r3), and 85% (DRISHT-GS1)
		RIM-ONE-r3	
		DRISHT-GS	
[34]	GoogleNet	HRF	Accuracy: 90% (HRF), 94.2% (RIM-ONE-r1), 86.2% (RIM-ONE-r2), and 86.4% (RIM-ONE-r3)
		RIM-ONE-r1	
		RIM-ONE-r2	
		RIM-ONE-r3	
[98]	U-Net	REFUGE	Accuracy: 93.4%
[99]	CNN with one layer (CNN1) CNN with two layers (CNN2)	RIM-ONE	Accuracy: 95.6% (CNN1) and 96.9% (CNN2)
			AUC: 98% (CNN1) and 97.8% (CNN2)
[100]	Transfer Learning GoogleNet Inception-V3	1542 images	Accuracy: 84.5%
			AUC: 93%
[101]	CNN with nineteen layers	48,116 images	Accuracy: 95.6%
			AUC: 98.6%
[102]	CNN with eighteen layers	1426 images	Accuracy: 98.1%
[32]	CNN with nineteen layers	ORIGA	Accuracy: 99.8% DICE: 87.2%
		DRIONS-DB	
		ONHSD	
		RIM-ONE	
[103]	Stack-U-Net	RIM-ONE-r3	IoU: 92%
			DICE: 96%
[104]	DC-GAN	MESSIDOR	AUC: 90.2%
		ONHSD	
		DRIVE	
		STARE	
		CHASE-DB	
		DRIONS-DB	
		SASTRA	
[105]	HDLS	1791 fundus photographs	Accuracy: 53% (optic cup), 12% (optic disc), and 16% (retinal nerve fiber layer defects)
[106]	DNN	2643 images	AUC: 94%
[107]	CNN Grad-CAM	SMC	Accuracy: 96%
			Sensitivity: 96%
			Specificity: 100%

## Data Availability

Not applicable.
